# Copper-Doped Porous Carbon Derived from Biomass Substrate: A High-Efficient Catalyst for the Thermal Decomposition and Combustion Performance of DAP-4

**DOI:** 10.3390/ijms27125251

**Published:** 2026-06-10

**Authors:** Yiming Wang, Jinchao Qiao, Qiang Zhou, Zichen Yan, Liwei Zhang

**Affiliations:** 1 National Key Laboratory of Solid Rocket Propulsion, Northwestern Polytechnical University, Xi’an 710072, China; 2 State Key Laboratory of Explosion Science and Safety Protection, Beijing Institute of Technology, Beijing 100081, China; 3 China Research and Development Academy of Machinery Equipment, Beijing 100089, China

**Keywords:** DAP-4, thermal decomposition, combustion, copper-doped porous carbon, catalysis mechanism

## Abstract

To address the urgent demand for eco-friendly and low-cost catalysts to replace toxic heavy-metal additives in energetic materials, this work focuses on developing biomass-derived copper-doped porous carbon (CuPC) as a high-efficiency catalyst for the thermal decomposition and combustion of molecular perovskite energetic material (H_2_dabco)NH_4_(ClO_4_)_3_(DAP-4). Biomass carbonaceous material has garnered extensive attention in many fields, owing to the low cost, high utilization efficiency, and environment protection. Herein, the CuPC catalysts were rationally designed and fabricated via the high-temperature carbonization treatment of biomass carbonaceous material precursor. The catalytic effects of CuPC on the thermal decomposition and combustion characteristics of DAP-4 were systematically investigated. The results revealed that CuPC possessed inherent catalysis property on the decomposition and combustion reaction of DAP-4. Cu_x_O_y_ nanoparticles were uniformly distributed on the surface of carbonized biomass substrates, endowing the catalysts with superior dispersibility. Thermal analysis results indicated that the addition of 5 wt% CuPC-3 reduced the thermal decomposition peak temperature from 378 °C of raw DAP-4 to 350 °C of DAP-4/CuPC-3. Moreover, the apparent activation energy of DAP-4 was notably decreased with the incorporation of CuPC catalysts. The combustion characterization results demonstrated that DAP-4 exhibited a more intense combustion process with the addition of CuPC, accompanied by elevated maximum combustion temperature and enhanced combustion heat. The catalytic mechanism of CuPC on the thermal decomposition and combustion of DAP-4 was further proposed. This work provides a targeted strategy for designing sustainable biomass-based catalysts to optimize the energy release performance of advanced molecular perovskite energetic materials.

## 1. Introduction

Biomass carbonaceous material had attracted much attention as an ideal precursor for the design and preparation of carbon-based materials due to its wide availability, low cost, renewable nature, and unique natural structure, which has greatly promoted the research and development of renewable and sustainable biomass materials [1,2,3,4,5,6]. Compared with traditional carbon materials derived from fossil resources, biomass-based carbonaceous materials possess adjustable porous structure, large specific surface area, high electrical conductivity, good chemical stability, and environmental friendliness, which had been widely studied in energy storage and conversion, electrocatalysis, environmental remediation, electromagnetic absorption, flexible electronics, and so on [7,8,9,10]. For example, flexible electrical circuits with carbonized flexible sheets of polyaramid were fabricated by a laser-induced carbonization method. The rough and highly porous microstructures with a higher degree of graphitization were obtained [11]. The bamboo-derived carbonaceous fiber with O, N-doping and hierarchical porous was fabricated by the combination of selective removal of hemicelluloses and lignin and plasma post-treatment, where the hierarchical porous structure is beneficial for an increase in electrolyte infiltration [12]. Porous lignin-based graphitic carbon with continuous three-dimensional porous framework was designed and used as promising biomass electrode materials, which could provide an effective platform for bulk charge transport in the composite electrodes [13]. The research showed an outstanding potential to the design and application of biomass carbonaceous material [14,15,16,17,18,19].

In the field of energetic materials, carbon-based materials are a class of important additives to modify the performance of explosives, and propellants [20,21,22,23,24,25]. For instance, Zhang et al. [26] prepared the carbon dot-based and carbon/metal catalysts, and used them to achieve great catalytic combustion enhancements. As is known to all, biomass carbonaceous materials, as a special and unique class of carbon materials, could also act as combustion catalysts, thermal stabilizers, conductive agents and dispersion carriers to regulate the thermal decomposition, combustion behavior, safety and energy release efficiency of traditional energetic components [21,27,28,29,30,31,32]. For instance, An et al. [33] reported that Fe/N-based biomass porous carbon was prepared simply by the high-temperature carbonization method with the biomass materials. Fe/N-based biomass porous carbon showed the enhanced thermal decomposition of molecular perovskite energetic materials. It was essential and significant to use biomass carbonaceous materials as additives in energetic materials because of their wide availability, low cost, and environmentally friendly characteristics.

Despite these advances, there remains a critical gap in developing biomass-derived catalysts specifically for molecular perovskite energetic materials (MPEMs) like DAP-4, which require efficient, low-toxicity additives to unlock their full potential in solid rocket propulsion. To bridge this gap, herein, biomass carbonaceous material was used as the carbonized substrates and precursors to design and prepare theCuPC catalysts via high-temperature carbonization. The microscopic morphology and structure of CuPC were characterized, and its catalytic effects on the thermal decomposition and combustion performance of DAP-4 were systematically investigated. Furthermore, the thermal decomposition and combustion mechanism of DAP-4 with CuPC additives were analyzed. This work aims to provide a novel strategy for the design and application of biomass carbon materials in the energetic field, addressing the urgent need for sustainable catalyst solutions to replace traditional heavy-metal additives.

## 2. Results and Discussion

### 2.1. Morphology and Structure

**Figure 1:**
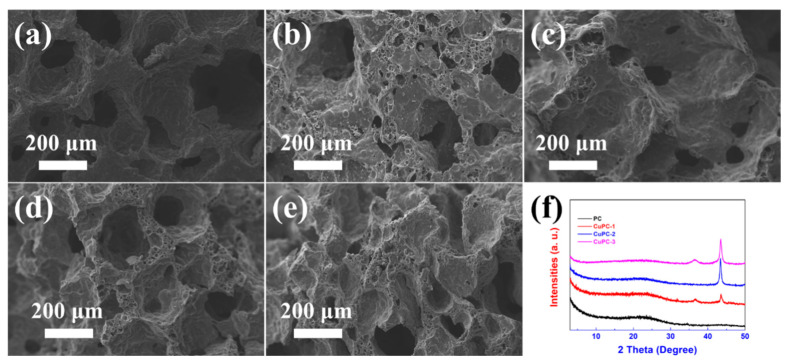
SEM images of (**a**) biomass substrate, (**b**) PC, (**c**) CuPC-1, (**d**) CuPC-2, (**e**) CuPC-3, and (**f**) its X-ray Diffraction (XRD) patterns.

The microscopic morphology and structure of biomass substrate, porous carbon (PC) and CuPC series catalysts was characterized, as shown in Figure 1, firstly. The Scanning Electron Microscope (SEM) images of biomass substrate, PC and CuPC series catalysts are displayed in Figure 1a–e. The original biomass substrate presents a loose and porous structure, which offers a favorable support for metal ion deposition.

After high-temperature carbonization, PC retains the hierarchical porous structure of the biomass precursor, with abundant micro/nano pores on the surface, which is conducive to the exposure of active sites and mass transfer. For CuPC-1, CuPC-2 and CuPC-3, with the increase of copper loading, no obvious agglomeration of copper-based particles is observed, indicating that Cu_x_O_y_ nanoparticles are uniformly dispersed on the porous carbon surface. This uniform dispersion is attributed to the anchoring effect of biomass carbon pores, which effectively inhibits the agglomeration of metal oxide nanoparticles during high-temperature carbonization.

**Figure 2:**
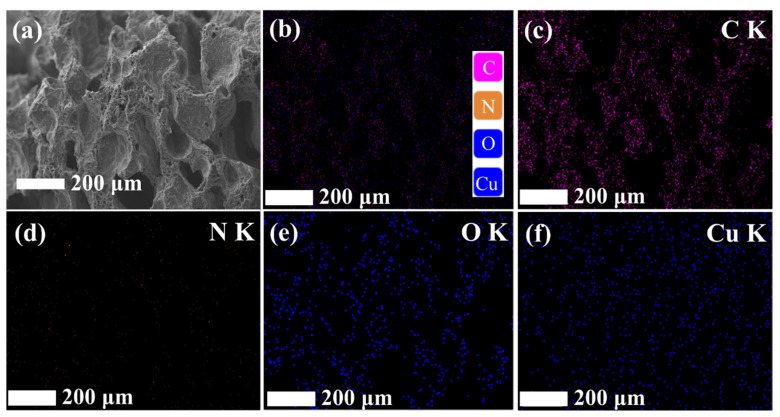
The SEM image of (**a**) CuPC-3, and (**b**–**f**) its corresponding EDX mappings.

The XRD patterns of PC and CuPC series catalysts are shown in Figure 1f. All samples exhibit a broad diffraction peak at around 23°, which is assigned to the (002) crystal plane of amorphous carbon, representing the typical characteristic of biomass-derived porous carbon. Additionally, a weak diffraction peak appears at approximately 43° in CuPC-1, CuPC-2 and CuPC-3 samples, and the peak intensity gradually strengthens with the increase of copper oxides loading. This peak is attributed to the (111) crystal plane of Cu_2_O or the (11-1) crystal plane of CuO, which are the main crystalline phases of Cu_x_O_y_ formed during high-temperature carbonization. For CuPC samples, no other obvious diffraction peaks of Cu_x_O_y_ are detected, which is mainly due to the low loading and high dispersion of Cu_x_O_y_ nanoparticles, resulting in weak overall crystallinity and only the relatively strong characteristic peak of Cu_x_O_y_ can be observed. The Energy Dispersive Spectrometer (EDS) mapping of CuPC-3 (Figure 2) further confirms the uniform distribution of C, N, O and Cu elements, proving that copper elements are successfully doped into the porous carbon matrix without obvious aggregation.

**Figure 3:**
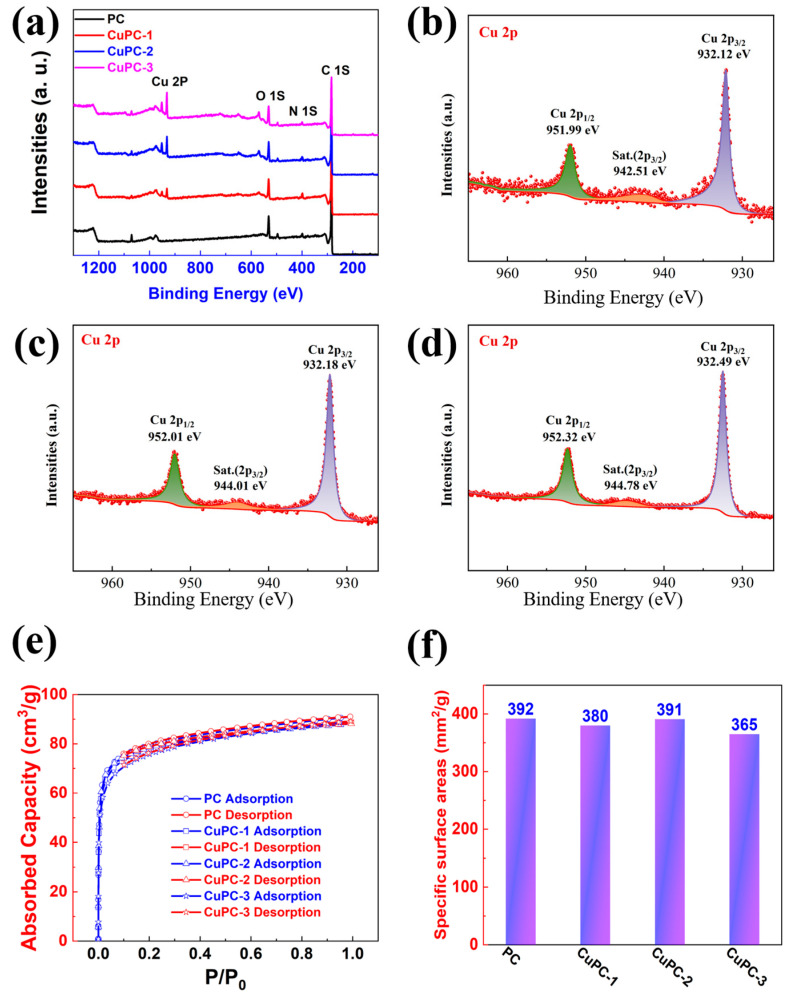
(**a**) XPS survey of CuPC, and Cu 2p XPS spectra of (**b**) CuPC-1, (**c**) CuPC-2, (**d**) CuPC-3. (**e**) BET curves, and (**f**) specific surface areas of samples.

The chemical composition and valence states of CuPC were analyzed by X-ray Photoelectron Spectroscopy (XPS), and the results are shown in Figure 3a–d. The full XPS survey spectrum (Figure 3a) reveals the presence of C 1s, N 1s, O 1s and Cu 2p peaks in CuPC samples, while no Cu signal is detected in PC. The high-resolution Cu 2p spectra (Figure 3b–d) show two characteristic peaks at ~932 eV and ~952 eV, corresponding to Cu 2p_3/2_ and Cu 2p_1/2_, respectively. The satellite peaks at ~942–944 eV confirm the existence of Cu^2+^ species, indicating that the copper component in CuPC is mainly Cu_x_O_y_ (CuO and Cu_2_O). The element contents of PC and CuPC series catalysts obtained by XPS and EDS are listed in Table 1. With the increase of copper nitrate concentration, the Cu content in CuPC gradually increases, while the C content decreases accordingly, which is consistent with the preparation design.

The Brunauer-Emmett-Teller (BET) adsorption–desorption curves and specific surface area of the samples are presented in Figure 3e,f. The specific surface areas are 392 mm^2^/g for PC, 380 mm^2^/g for CuPC-1, 391 mm^2^/g for CuPC-2, and 365 mm^2^/g for CuPC-3. All samples exhibit typical type IV isotherms with H_4_ hysteresis loops, indicating the existence of mesoporous structures. The specific surface area of PC is the highest, and the specific surface area of CuPC decreases gradually with the increase of copper loading, for the reason that the deposited Cu_x_O_y_ nanoparticles block part of the micro/nano pores of the biomass carbon, which is consistent with the previous morphology analysis. Despite the reduction in specific surface area, CuPC still maintains a rich porous structure, which is beneficial to the catalytic reaction between the catalyst and DAP-4.

### 2.2. Thermal Decomposition Properties

**Figure 4:**
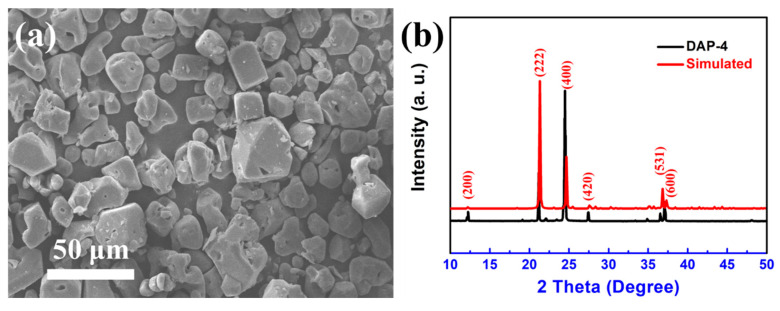
(**a**) SEM image and (**b**) XRD pattern of DAP-4.

The SEM image and XRD pattern of the synthesized DAP-4 are shown in Figure 4. The as-obtained DAP-4 sample presents a cubic-like morphology. The XRD diffraction peaks are consistent with the standard card (CCDC: 1528106) [34]. As shown in Figure 4b, the diffraction peaks at 12.6°, 21.8°, 25.2°, 27.5°, 37.0°, and 37.8° are reflected from the crystal planes of (200), (222), (400), (420), (531), and (600). The characterization results proved the successful preparation of high-purity DAP-4.

**Figure 5:**
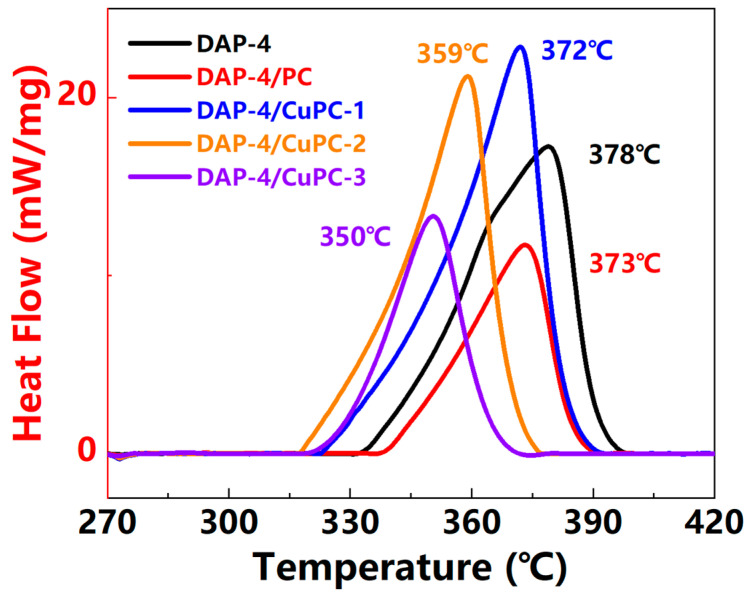
Differential Scanning Calorimetry (DSC) curves of raw DAP-4, DAP-4/PC and DAP-4/CuPC-X composites.

The DSC curves of raw DAP-4 and DAP-4/CuPC composites at a heating rate of 10 °C/min are illustrated in Figure 5. Raw DAP-4 presents a sharp thermal decomposition peak at 378 °C, with a single decomposition stage. The addition of pure PC exerts little effect on the decomposition peak temperature of DAP-4, indicating that PC possesses negligible catalytic activity. In contrast, the incorporation of CuPC catalysts significantly reduces the thermal decomposition peak temperature of DAP-4, and the reduction effect is enhanced with the increase of copper loading. Specifically, the decomposition peak temperature of DAP-4/CuPC-3 is reduced to 350 °C, which is 28 °C lower than that of raw DAP-4, demonstrating the excellent catalytic activity of CuPC.

**Figure 6:**
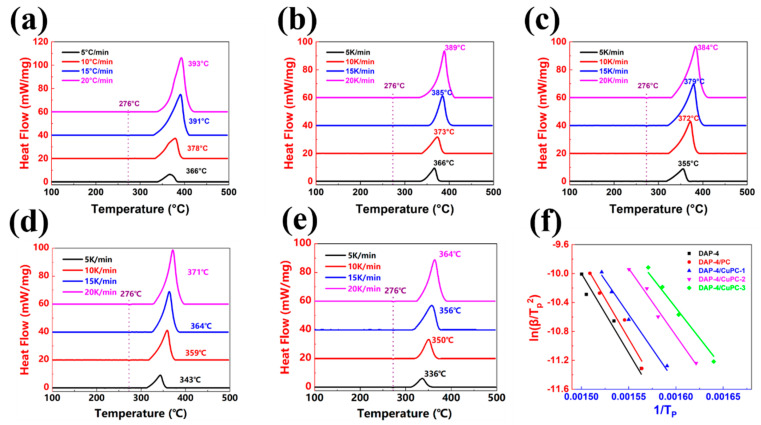
DSC curves of (**a**) raw DAP-4, (**b**) DAP-4/PC, (**c**) DAP-4/CuPC-1, (**d**) DAP-4/CuPC-2, (**e**) DAP-4/CuPC-3 with different heating rates (5, 10, 15, and, 20 °C/min), and (**f**) linear fitting result of ln(*β*/*T_p_*^2^) versus 1/*T_p_* for the fitted data.

To further explore the thermal decomposition kinetics of DAP-4/CuPC composites, DSC tests at different heating rates (5, 10, 15, 20 °C/min) were carried out (Figure 6a–e). The thermal decomposition peak temperature of all samples increases linearly with the increase in heating rate, which is a typical thermal analysis phenomenon. The apparent activation energy ($E_{a}$) was calculated by the Kissinger equation (Equation (1)):

(1) $$\ln\frac{\beta }{{T_{p}}^{2}}=\ln\frac{AR}{E_{a}}-\frac{E_{a}}{RT_{p}}$$

where β is the heating rate (°C/min), $T_{p}$ is the thermal decomposition peak temperature (K). R is the gas constant of 8.314 J/(mol·K), $E_{a}$ is the apparent activation energy (kJ/mol), and A is the pre-exponential factor.

The linear fitting curve of $\ln\left(\beta /T_{p}^{2}\right)$ versus 1/$T_{p}$ is shown in Figure 6f, and the kinetic parameters are listed in Table 2. The apparent activation energy of raw DAP-4 is 178.9 kJ/mol, while that of DAP-4/PC is slightly increased to 181.1 kJ/mol. With the addition of CuPC-1, CuPC-2 and CuPC-3, the $E_{a}$ values decrease to 154.7 kJ/mol, 151.4 kJ/mol and 146.1 kJ/mol, respectively. The significant reduction in activation energy indicates that CuPC catalysts effectively reduce the energy barrier of DAP-4 thermal decomposition, thereby accelerating the decomposition reaction.

To delineate the catalytic merit of CuPC, its performance is compared with the most relevant Cu-based systems documented for DAP-4 and ammonium perchlorate (APC) (Table 3).

First, bare nano-CuO demonstrates high intrinsic activity. Bhaskaran et al. [35] reported that 5 wt% nano-CuO reduces the DAP-4 decomposition peak by up to 61 °C (e.g., from ~364 to 303 °C) and elevates the decomposition enthalpy from 3496 to 4256 J·g^−1^, with the apparent activation energy ($E_{a}$) dropping from ~331 to 216 kJ·mol^−1^ (Kissinger method). Similarly, Shukri Ismael et al. [36] showed that 1 wt% Cu nanoparticles (50 nm) shift the DAP-4 peak from ~399 to ~352.5 °C (Δ ≈ 47 °C) and slash $E_{a}$ from 216 to 135 kJ·mol^−1^. These studies confirm the excellent redox capability of Cu species, driven by their variable oxidation states (Cu^0^/Cu^+^/Cu^2+^) and facilitation of proton transfer from H_2_dabco^2+^/NH_4_^+^ to ClO_4_^−^.

Second, CuPC-3 (this work, 5 wt%) delivers a 28 °C shift (378 → 350 °C) and a 32.8 kJ·mol^−1^ $E_{a}$ reduction (178.9 → 146.1 kJ·mol^−1^). While its per-gram activity is lower than that of bare CuO/Cu nanoparticles (NPs), CuPC resolves the critical limitation of naked oxides: agglomeration. Refs. [37,38] note that bare CuO/Cu NPs require careful dispersion (e.g., solvent evaporation in Ref. [38]) to avoid clustering, whereas CuPC anchors Cu_x_O_y_ nanoparticles within the hierarchical pores of biomass carbon (Figure 1 and Figure 2), ensuring stable, uniform distribution even at 5 wt% loading. Additionally, the carbon matrix enhances condensed-phase heat transfer and gaseous-product diffusion—benefits absent in oxide-only systems.

Third, contextual APC benchmarks further clarify the niche of CuPC. For APC, Elbasuney & Yehia [37] demonstrated that 1 wt% colloidal CuO (~15 nm) collapses APC’s two exothermic stages into a single peak at 350.1 °C and increases total heat release by 53% (from ~938 to 1268.4 J·g^−1^). Such APC studies confirm the general aptitude of Cu-based surfaces to accelerate ClO_4_^−^-involved redox; however, DAP-4’s intracrystalline perovskite pathway (proton transfer within the NH_4_^+^/H_2_dabco^2+^/ClO_4_^−^ lattice) represents a mechanistically distinct, more “solid-state” catalysis problem—precisely why interfacial contact and oxide dispersion (as in CuPC) matter.

**Table 3:** Comparative catalytic effects of various catalysts on the thermal decomposition of DAP-4 (or AP for contextual reference).

System (Loading)	Target	Conditions	T_p_(Pure) → T_p_(Cat)/°C	ΔT_p_/°C	E_a_(Pure) → E_a_(Cat)/kJ·mol^−1^	Key Advantage
DAP-4/5 wt% CuPC-3 (this work)	DAP-4	~50 μm, 10 °C·min^−1^, N_2_	378 → 350	−28	178.9 → 146.1	Cu_x_O_y_ pore-confined in biomass carbon; low-cost one-step synthesis; no heavy metals
DAP-4/5 wt% nano-CuO (Ref. [37])	DAP-4	Co-precipitated CuO, TG-DSC 5 °C·min^−1^, Ar	~364 → 303	−61	~331 → ~216	High intrinsic activity; oxide-only → agglomeration risk during formulation
DAP-4/1 wt% Cu NPs (Ref. [38])	DAP-4	50 nm Cu NPs, solvent-evaporation mixing, DSC 10 °C·min^−1^, N_2_	~399 → ~352.5	≈−47	~216 → ~135	Reactive Cu fuel + in situ Cu → CuO; excellent ignition, but requires dispersion control
DAP-4/5 wt% Fe/N-biomass carbon (Ref. [33])	DAP-4	Fe/N-doped porous carbon	~384 → ~305	−79	—	Strong shift via N-doping synergy; CuPC simpler (no extra dopants)
APC/1 wt% CuO (Ref. [39])	APC	Colloidal CuO (~15 nm), DSC 5 °C·min^−1^, N_2_	Two exotherms → single peak 350.1	—	—	Confirms CuO’s hallmark effect on APC (HTD collapse +53% heat release); not a molecular-perovskite comparison
HMX-CMDB/0.65 wt% CDs/Cu (Ref. [26])	HMX-CMDB	CDs/Cu composite (oxidative etching + hydrothermal), target-line method 4–18 MPa	Decomposition temperature decreased by 9.2 °C	—	35.9 kJ·mol^−1^ reduction (Kissinger	Synergistic carbon–Cu effect; but requires synthetic carbon dots + multi-step processing

### 2.3. Combustion Performances

**Figure 7:**
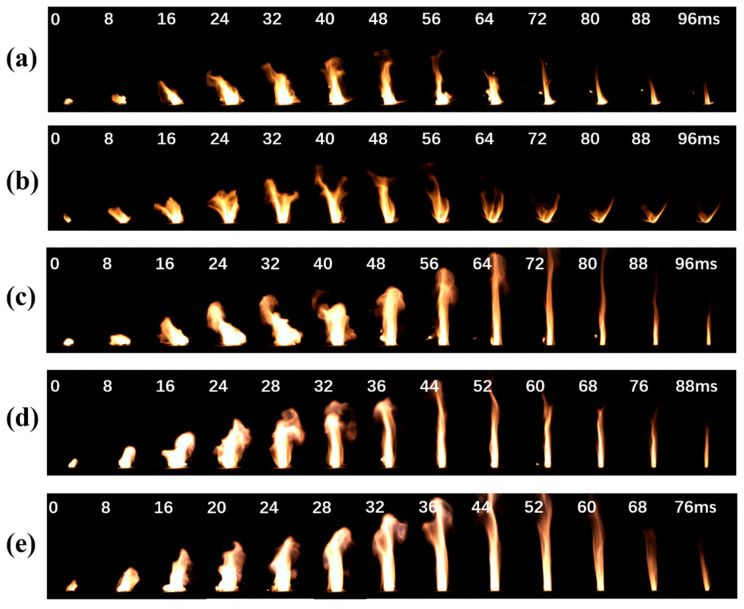
The ignition and combustion processes of (**a**) DAP-4, (**b**) DAP-4/PC, (**c**) DAP-4/CuPC-1, (**d**) DAP-4/CuPC-2, and (**e**) DAP-4/CuPC-3.

The ignition of DAP-4 and DAP-4/CuPC composites was initiated using a Ni-Cr heating wire (diameter: 0.3 mm). All combustion tests were conducted in ambient air at atmospheric pressure (0.1 MPa), simulating realistic conditions for energetic oxidizer ignition measurements. The ignition and combustion processes of raw DAP-4 and DAP-4/CuPC composites were recorded by high-speed photography, as shown in Figure 7. Raw DAP-4 can achieve self-sustaining combustion after ignition, with a mild combustion flame and a long combustion duration. The combustion flame of DAP-4/PC is weaker and the duration is longer, due to the consumption of oxidizing components by pure carbon. In contrast, DAP-4/CuPC composites show more intense combustion flames, brighter flame color and shorter combustion duration, and the combustion intensity increases with the increase of copper loading. DAP-4/CuPC-3 exhibits the most violent combustion, with a large flame plume and rapid energy release, indicating that CuPC effectively promotes the combustion reaction of DAP-4.

**Figure 8:**
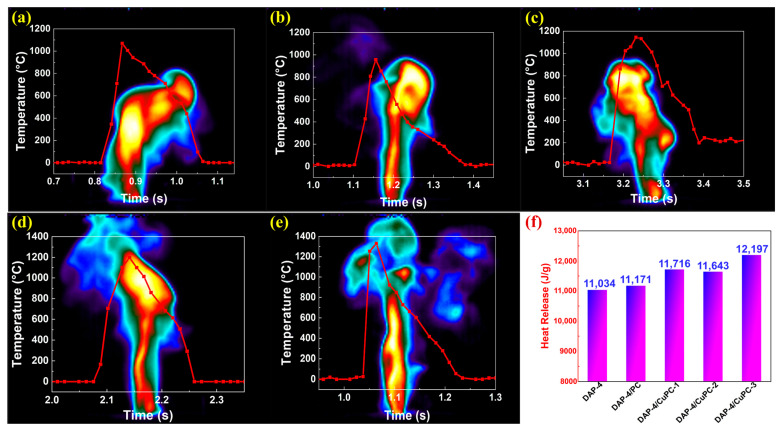
The maximum combustion temperature curves and relevant infrared imaging of (**a**) raw DAP-4, (**b**) DAP-4/PC, (**c**) DAP-4/CuPC-1, (**d**) DAP-4/CuPC-2, and (**e**) DAP-4/CuPC-3. (**f**) Combustion heat value of samples.

The maximum combustion temperature and combustion heat of the samples are presented in Figure 8. The maximum combustion temperature and combustion heat of raw DAP-4 are relatively low. The addition of PC reduces the combustion temperature and heat release, while the incorporation of CuPC significantly increases both parameters. DAP-4/CuPC-3 has the highest maximum combustion temperature and combustion heat, which is attributed to the synergistic effect between Cu_x_O_y_ nanoparticles and porous carbon: Cu_x_O_y_ accelerates the redox reaction between fuel cations and oxidant anions in DAP-4, while porous carbon provides a conductive network and promotes mass transfer, thus enhancing the energy release efficiency of DAP-4.

**Figure 9:**
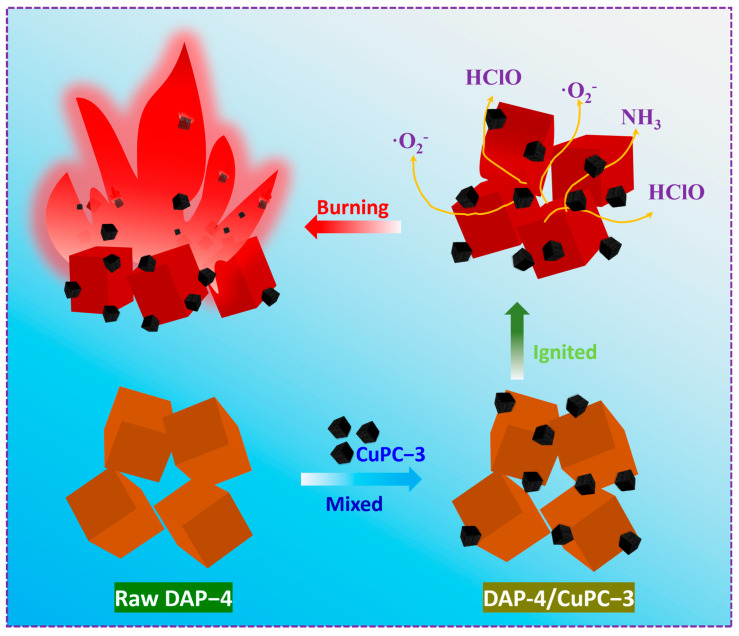
Scheme diagram of the role of CuPC-3 in the thermal decomposition and combustion processes of DAP-4.

Based on the above characterization and performance tests, the catalytic thermal decomposition and combustion mechanism of DAP-4 over CuPC is proposed in Figure 9. DAP-4 has a stable ABX_3_ perovskite structure, with strong intermolecular hydrogen bonding and Coulombic force, leading to a high thermal stability. When CuPC is added, the uniformly dispersed Cu_x_O_y_ nanoparticles on the porous carbon surface act as active sites, which can adsorb and activate the perchlorate anions and fuel cations in DAP-4. Under heating conditions, Cu_x_O_y_ promotes the hydrogen transfer from NH_4_^+^ and H_2_dabco^2+^ to ClO_4_^−^ [38,39,40,41], accelerating the cleavage of chemical bonds and the formation of intermediate products such as NH_3_, HClO_4_ and dabco. Meanwhile, the porous carbon substrate provides a large contact area and accelerates the diffusion of gas products, further promoting the thermal decomposition of DAP-4. During the combustion process, the catalytic effect of Cu_x_O_y_ reduces the ignition energy of DAP-4, triggers a rapid redox reaction, and realizes efficient and intense energy release, thus improving the combustion performance of DAP-4.

The intensified, shorter-duration combustion of DAP-4/CuPC composites aligns with the broadly reported ability of Cu-based species to accelerate ClO_4_^−^-associated redox sequences (Refs. [26,39]). The key advantage of CuPC over the most active bare-oxide benchmarks lies in formulation robustness. Refs. [37,38] confirm that nano-CuO and Cu NPs deliver larger single-parameter shifts (Δ*T_p_* up to −47…−61 °C) in lab-scale DAP-4 blends; however, those systems expose naked oxide/metal surfaces that agglomerate during mixing and lack an internal conductive scaffold. By contrast, CuPC couples dispersed Cu_x_O_y_ active sites with a hierarchical, thermally percolating biomass-carbon host, so the composite’s combustion still registers a higher *T_max_* and higher calorimetric heat release than DAP-4/PC (which, being inert carbon, can even dilute oxygen availability and depress combustion metrics).

From an application standpoint, CuPC offers a low-toxicity, heavy-metal-free alternative to legacy Pb/Cr burn-rate modifiers. Zhang et al. [26] recently demonstrated that carbon–dot/Cu (CDs/Cu) composites, when replacing carbon black in HMX-CMDB propellants, reduce the decomposition temperature by 9.2 °C, lower $E_{a}$ by 35.9 kJ·mol^−1^, shorten laser ignition delay by 67.5 ms, and boost the burning rate from 4.61 to 13.41 mm·s^−1^ at 4 MPa. While CDs/Cu achieve excellent performance via synergistic carbon–Cu effects, it relies on synthetic carbon dots and multi-step hydrothermal processing. CuPC, by contrast, utilizes a food-grade biomass scrap and a single-step 700 °C carbonization, completely avoiding functionalization, exfoliation, or noble-metal-free routes. Relative to other biomass-derived catalysts for DAP-4 (e.g., Fe/N-doped porous carbon, Ref. [33]), CuPC trades some ultimate Δ*T_p_* for a cleaner, more scalable chemistry and a Cu-centered redox activity that directly couples to the perchlorate pathways highlighted in the DAP-4 literature (Refs. [37,38]).

## 3. Materials and Methods

### 3.1. Chemicals and Materials

Raw AP (NH_4_ClO_4_) solid microparticles were supplied by Henan Nayu Co., Ltd. (Xinxiang, China), and used directly without further purification. Commercial perchloric acid (HClO_4_, 70%), Triethylenediamine (dabco, C_6_H_12_N_2_), and Cu(NO_3_)_2_·3H_2_O were purchased from Shanghai Aladdin Biochemical. Technology Co., Ltd. (Shanghai, China).

DAP-4 micro particle samples (~50 μm) were synthesized via one-pot molecular assemble method [34,42,43]. A molar ratio of AP:HClO_4_:C_6_H_12_N_2_ = 1:2:1 was selected based molecular perovskite ABX_3_. In a synthesis experiential, 0.25 mmol AP, 0.25 mmol dabco, and 0.408 mL HClO_4_ solution (0.2 mmol) were added into 50 mL deionized water together and dissolved completely at room temperature for 24 h. The DAP-4 samples were obtained by re-crystallization at room temperature.

**Figure 10:**
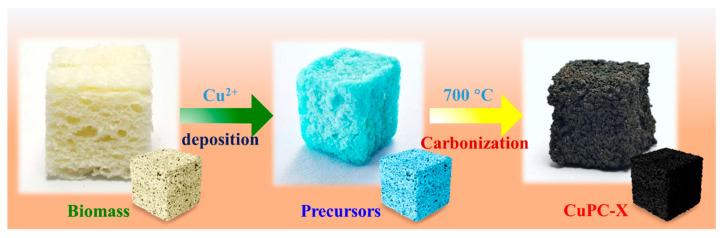
The scheme of preparation process of CuPC catalysts.

Biomass steamed bun materials, selected as the carbonized precursor, were obtained from a local market, which was prepared via flour fermentation and traditional steaming process. Wheat flour, water, and dry yeast were uniformly mixed in a 2 L beaker to form a smooth dough. The dough was kneaded for 10 min, then fermented for 60 min until its volume approximately doubled. The biomass steamed bun, as shown in Figure 10, was obtained by fermented precursors steamed with boiling water for 20 min.

CuPC-X series catalysts were fabricated by the combination of metal ion deposition and high-temperature carbonization method, as illustrated in Figure 10. Firstly, copper nitrate aqueous solutions with concentrations of 0.03 g/mL, 0.06 g/mL and 0.09 g/mL were configured, respectively. The biomass steamed bun was cut into small pieces and immersed in the above copper nitrate solutions for 12 h to ensure sufficient infiltration of copper ions. Then, the impregnated precursors were dried in a vacuum oven at 80 °C for 12 h to remove excess water. Finally, the dried precursors were transferred to a tube furnace and carbonized at 700 °C for 3 h under nitrogen atmosphere, with a heating rate of 5 °C/min. For comparison, pure PC without copper doping was prepared under the same carbonization conditions. The obtained samples were denoted as CuPC-1, CuPC-2 and CuPC-3 corresponding to the incremental concentration of copper nitrate solution.

The DAP-4/CuPC composite samples for thermal decomposition and combustion tests were prepared via simple physical mixing. A predetermined mass ratio of DAP-4 and CuPC (5 wt% CuPC) was blended using an agate mortar and pestle for 30 min to ensure uniform dispersion. This method simulates the conventional additive incorporation process in solid propellant manufacturing, avoiding interference from solvent residues or unintended chemical interactions.

### 3.2. Sample Characterization

The crystal phase structure of the samples was characterized by X-ray diffraction (XRD, Rigaku, Tokyo, Japan) with Cu Kα radiation (λ = 1.5406 Å). The microscopic morphology and element distribution were observed via a field-emission scanning electron microscope (SEM, Carl Zeiss Microscopy GmbH, EVO 10 MA, Jena, Germany) equipped with an energy dispersive X-ray spectrometer (EDS), operated at an accelerating voltage of 10 kV. The chemical valence states of elements were analyzed by a thermo fisher ESCALAB 250 X-ray photoelectron spectroscopy (XPS, Thermo Fisher Scientific, Richardson, TX, USA). The specific surface area and pore structure were measured by a Brunauer–Emmett–Teller (BET) specific surface area analyzer (CIQTEK Climber, CIQTEK Co., Ltd., Hefei, Anhui, China). Thermal decomposition behavior was characterized by a simultaneous TG-DSC analyzer (STA 449 F3, NETZSCH-Gerätebau GmbH, Selb, Bavaria, Germany) in nitrogen atmosphere, with a temperature range of 100–550 °C and heating rates of 5, 10, 15, 20 °C/min. The ignition and combustion process was recorded by a high-speed video camera (Phantom M340, Vision Research Inc., Wayne, NJ, USA) at 1000 frames per second, and the combustion temperature was tested by an infrared thermometer (X6520sc, Teledyne FLIR LLC, Wilsonville, OR, USA). The combustion heat was measured by a closed combustion bomb under inert gas environment, with a sample mass of 0.1 g.

## 4. Conclusions

In this work, to address the lack of eco-friendly catalysts for molecular perovskite energetic materials, a series of CuPC catalysts were successfully prepared via a green and facile high-temperature carbonization method using biomass steamed bun as the precursor. The effects of CuPC on the thermal decomposition and combustion performance of energetic molecular perovskite DAP-4 were systematically studied. The results show that Cu_x_O_y_ nanoparticles are uniformly dispersed on the surface of biomass porous carbon, and CuPC exhibits excellent catalytic activity for DAP-4. With the addition of 5 wt% CuPC-3, the thermal decomposition peak temperature of DAP-4 is reduced by 28 °C, and the apparent activation energy is decreased from 178.9 kJ/mol to 146.1 kJ/mol. Moreover, CuPC significantly enhances the combustion intensity of DAP-4, shortens the combustion duration, and increases the maximum combustion temperature and combustion heat release. The catalytic mechanism is attributed to the activation effect of Cu_x_O_y_ active sites on chemical bonds and the synergistic promotion of porous carbon on mass transfer.

Furthermore, this work provides several promising prospects for future research and practical applications.

First, the CuPC catalyst design can be extended to other molecular perovskite energetic materials beyond DAP-4, such as halogen-free variants, to tailor energy release profiles for diverse operational requirements.

Second, CuPC offers a low-toxicity, biomass-derived alternative to traditional heavy-metal catalysts in ammonium perchlorate-based composite propellants, contributing to the development of greener energetic formulations.

Third, the use of low-cost biomass precursors and simple carbonization processes highlights the scalability of CuPC for industrial production, with potential integration into next-generation solid rocket propulsion systems or pyrotechnic devices requiring high energy density and controlled combustion.

Finally, future studies could employ in situ characterization techniques to dynamically track the interactions between Cu_x_O_y_ nanoparticles and energetic material lattices, further clarifying the role of biomass-derived carbon matrices in stabilizing reactive intermediates during decomposition.

This work successfully demonstrates that biomass-derived CuPC catalysts can effectively replace toxic heavy-metal catalysts, offering a sustainable pathway for enhancing the energy release efficiency of advanced energetic materials and advancing the practical application of molecular perovskite energetic materials in aerospace and defense fields.

## Figures and Tables

**Table 1:** Elemental composition (wt%) of PC and CuPC-X samples determined by XPS and EDX.

	XPS Results (wt%)				EDX Results (wt%)			
	C	N	O	Cu	C	N	O	Cu
PC	84.85	3.62	9.66	0	81.81	3.55	14.63	0
CuPC-1	83.85	4.50	9.95	0.97	78.43	2.39	3.41	15.77
CuPC-2	82.69	5.28	9.60	1.71	71.56	0.03	5.65	22.76
CuPC-3	77.56	4.32	13.30	2.81	62.61	0.36	6.62	30.41

**Table 2:** Kinetic parameters of thermal decomposition of DAP-4 and DAP-4/CuPC.

	$T_{p}$ (°C)				$E_{a}$ (kJ/mol)	logA
	5 °C/min	10 °C/min	15 °C/min	20 °C/min		
Raw DAP-4	366	378	391	393	178.9	14.3
DAP-4/PC	366	373	385	389	181.1	14.59
DAP-4/CuPC-1	355	372	379	384	154.7	12.49
DAP-4/CuPC-2	343	359	364	371	151.4	12.48
DAP-4/CuPC-3	336	350	356	364	146.1	11.29

## Data Availability

The original contributions presented in this study are included in the article. Further inquiries can be directed to the corresponding author.
